# Vitamin D Fortification: A Promising Approach to Overcome Drug Resistance and Tolerance in Therapeutic Interventions

**DOI:** 10.1155/2024/9978076

**Published:** 2024-11-22

**Authors:** Digvijaya, Saurabh Mittal, Prateek Mittal, Ruhi Singh, Simran Gupta, Taniya Singh, Rahmuddin Khan, Nafaa Alzobaidi, Abdulsalam Alhalmi

**Affiliations:** ^1^Department of Pharmacology, School of Pharmaceutical Education and Research, Jamia Hamdard, New Delhi 110062, India; ^2^Center of Pharmaceutics, Amity Institute of Pharmacy, Amity University, Noida 201303, Uttar Pradesh, India; ^3^Department of Dentistry, Naina Devi Dental Care, New Delhi 110062, India; ^4^Department of Pharmaceutics, School of Pharmaceutical Education & Research, Jamia Hamdard, New Delhi 110062, India; ^5^Department of Pharmacology, College of Pharmacy, University of Aden, Aden, Yemen; ^6^Department of Pharmaceutics, College of Pharmacy, University of Aden, Aden, Yemen

**Keywords:** drug resistance, drug therapy failure, drug tolerance, resistance, tolerance, vitamin D

## Abstract

The issue of drug resistance and tolerance presents a significant challenge as it diminishes the efficacy and potency of medications, posing a formidable obstacle for physicians striving to enhance pharmacological therapy worldwide. These resistance mechanisms can arise from genetic predispositions or as a consequence of medical interventions. Notably, acquired resistance or tolerance may extend to other drugs within the same or different classes, despite differing mechanisms of action. This phenomenon leads to the ineffectiveness of various pharmacological treatments over time, hindering the attainment of complete remission for numerous illnesses spanning metabolic disorders, autoimmune diseases, carcinomas, infectious diseases, cardiovascular diseases, and neurological disorders. Vitamin D, an essential lipid-soluble nutrient crucial for regulating calcium and phosphorus levels, is emerging as a potential solution to counteract treatment resistance and tolerance in various conditions such as cancer, tuberculosis, and depression. This review scrutinizes existing research and offers insights for future investigations aimed at fully elucidating the therapeutic potential of vitamin D in mitigating the challenges associated with prolonged medication regimens and drug treatment failures.

**Trial Registration:** ClinicalTrials.gov identifier: NCT01169259 and NCT04342598.

## 1. Introduction

Drug resistance and tolerance and its increasing rate are major concerns of the health care system as they limit the efficacy and potency of the drugs and play a crucial part in delaying the treatment of various ailments. The concern of drug resistance is universal and clinicians worldwide are struggling to overcome the issue to offer better pharmacological treatment to the patients. Thus, identifying a safe and effective agent that could reverse the resistance mechanism is the necessity of the times. The phenomenon of drug resistance was initially documented in 1947, when certain bacteria demonstrated the ability to develop resistance against the previously successful antibiotic known as penicillin [1]. It was subsequently characterized as the diminished effectiveness of a medication in addressing an illness or ailment. Drug resistance is reported more commonly in antimicrobial agents, anticancer agents, antitubercular drugs, antiparasitic agents, etc. [2]. Majorly, there are cellular, molecular, genetic, and clinical factors that are found to contribute to the emergence of drug resistance.

The resistance can be inherited or acquired during the treatment of a disease. The major drawback of the acquired resistance or tolerance is that it can also lead to cross-resistance towards other drugs from either the same or different class and also that might have altogether a different mechanism of action. Intrinsic or inherited drug resistance is attributed to the evolutionary genetic changes in the organisms, while gene transfer, amplification, and alterations result in modified protein expression [3]. Mutations or evolutionary changes leading to intrinsic drug resistance can occur due to many reasons, namely, the lack or moderation of the drug's target site; activation of efflux pumps for drugs, inactivation of the drug by modification or degradation; high detoxification capacity of microorganisms, the low drug delivered to the target site; the rate of cell division as in case of resistance towards anticancer agents; chemically instigated adaptive alterations; and genetic reflexes/response to stress [4, 5]. Acquiring resistance to a drug is developed on long-term use of a drug and is associated with the changes in the organization of the genetic material and the take-up of exogenous DNA to modify the integral genetic makeup. Pathogens acquire resistance mostly when there is at least one microorganism with resistant traits amongst a group of heterogeneous microorganisms, i.e., a superbug pathogen that replicates with the new and modified genetic composition resulting in a resistant population of microorganisms. The resistance developed by microbes towards a drug has its genetic basis which can be altered by knocking out or introducing the gene, thus altering the overall cellular gene expression [6]. Wherein the intrinsic resistance is attributed to the inherent characteristics of chromosomes having genes and the efflux system; the acquired resistance is attributed to the altered gene transfer occurring via processes such as transformation, transduction, or conjugation resulting in genetic mutations [7].

Drug tolerance was first elucidated in 1998 [8] and is defined as the reduced response of an organism towards a certain dose of a drug on repeated or prolonged usage. The higher dose of the drug can then be used to produce a similar effect which though can escalate tolerance but further diminishes the efficacy of the drug. Drug tolerance can be reversed by discontinuation of the drug for some time. Drug tolerance can be pharmacokinetic, behavioral, or pharmacodynamics [9]. Pharmacokinetic tolerance is due to the decreased levels of the bioavailable drug on repeated or prolonged usage of the drug. This could be due to the altered drug distribution in various compartments of the body or changes in metabolism possibly due to hepatic microsomal enzyme induction or autoinduction or increased excretion. Many drugs are capable of inducing their metabolism or excretion. Moreover, the pharmacokinetic tolerance is usually not limited to the drug inducing the microsomal enzymes, thus it results in developing cross-tolerance amongst the pharmacologically similar drugs [10]. Behavioral tolerance is defined as the establishment of tolerance towards the behavioral effects of the drug. This has been demonstrated as the development of tolerance to ethanol due to intoxicated practice [11]. However, the extent of translation of the phenomena of behavioral tolerance from preclinical findings to the clinics is not clear. Pharmacodynamic tolerance is explained as the altered pharmacological properties of the drug on repeated or prolonged use which is attributed to the transformations in the organ systems or pathways due to drug-receptor(s) interaction. The major factors involved are the dosage, duration, and frequency of drug administration [12]. As the pharmacodynamic tolerance develops due to the adaptive changes developing in some pathways or receptors, it affects all the drugs operating on a similar mechanism and hence results in cross-tolerance [13].

Amidst the growing global worry over medication resistance and tolerance, young researchers are dedicated to discovering a safe and effective solution that can reverse the resistance mechanism. This is an urgent issue that needs to be addressed in order to properly treat patients. The truth remains, however, that numerous vitamins and bioactive substances have demonstrated promise in overcoming medication tolerance and resistance via a variety of pathways. Curcumin, resveratrol, and quercetin are bioactive compounds that have been investigated for their potential to suppress P-glycoprotein overexpression and other drug-resistance pathways in cancer cells [5]. In addition, oxidative stress is commonly linked to drug resistance; vitamins C and E, which have antioxidant qualities, can help combat this. Furthermore, research suggests that vitamins, such as vitamin D, can influence immune responses, which could make some medications work better. We hope to summarize vitamin D's ability to reverse medication resistance and tolerance in this study [14]. This study aims to summarize vitamin D's capability to reverse medication resistance and tolerance.

Since its discovery as an antirachitic component and its synthesis via ultraviolet irradiation in the 1920s, vitamin D, also known as the “sunshine vitamin,” has seen a meteoric rise in popularity over the past decade. While rickets began to decline as a major medical issue in the industrialized world in the following decade, vitamin D research waned and hit a low point in the middle of the twentieth century when supplementation was blamed for idiopathic hypercalcemia [14]. The connection between vitamin D and metabolic bone disease had been forgotten until the endocrine system for vitamin D was discovered in 1970. A number of other medical fields have begun investigating vitamin D and its effect after it was discovered that the vitamin D hormone operates through a nuclear receptor [15]. This has sparked a flurry of research into vitamin D's potential effects on conditions such as arthritis, heart disease, infections, cancer, and granuloma formation. Many modern clinical researchers, basing their conclusions on the interpretation of epidemiological measurements, have proposed that high supplemental vitamin D intake may have a role in the prevention of numerous diseases [14, 16].

Though various studies suggest the implication of vitamin D in treating diseases, yet a lot more research studies are required to conclude the fact. Herein, we are just trying to highlight and summarize the research studies that can help perceive the fact that vitamin D might also prove to be effective and protective in the development of drug resistance or tolerance and hence can be repurposed for the reversal of drug resistance and tolerance.

## 2. Mechanisms of Drug Resistance in Various Ailments

The study examines the molecular mechanisms underlying the development of drug resistance and tolerance. It emphasizes the critical importance of this research area in understanding how organisms adapt to medications. The section explores various cellular and molecular processes, including genetic mutations, changes in gene expression, and cellular responses. These mechanisms collectively enable organisms to withstand the effects of drugs over time. By deepening our understanding of these processes, there is potential to develop more effective strategies to counteract medication resistance and tolerance, thereby improving treatment outcomes.

### 2.1. Depression

Antidepressant drugs play a significant role in the treatment of major depressive disorders. While antidepressant medication may improve biological vulnerability to depression, it can also increase the severity of depressive symptoms and decrease the effectiveness of pharmaceutical treatment if used over an extended period of time [17]. Antidepressant drug discontinuation results in a relapse of the condition and also recurrent depression is found to be less responsive to the pharmacological treatment with antidepressants [18]. The following factors are probably responsible for the development of tolerance: pharmacological tolerance, ineffective placebo, worsening of disease, altered and progressive pathogenesis, insufficient metabolite accumulation, unidentified rapid cycling, and nonresponsive prophylactic treatment [19–21].

The process of developing tolerance to antidepressant medicines, namely, those that affect serotonin (5-HT) receptors, involves multiple intricate biochemical pathways. The mechanisms of action can differ based on the specific antidepressant and the individual's treatment response. Here is an elaborate elucidation: (1) Decrease in the expression or activity of 5-HT receptors: Extended usage of antidepressants can result in a reduction in the quantity or responsiveness of 5-HT receptors in the brain. The downregulation takes place as a compensatory reaction to the elevated levels of serotonin induced by the medication. Consequently, the brain's responsiveness to serotonin decreases, leading to a decrease in the effectiveness of the antidepressant. (2) Desensitization of 5-HT receptors: Prolonged exposure to antidepressants can cause the desensitization of 5-HT receptors, in addition to downregulation. Desensitization is the process in which the repetitive stimulation of receptors leads to a decrease in their sensitivity to neurotransmitters. This can enhance the development of tolerance by diminishing the capacity of serotonin to bind to and stimulate its receptors. (3) Modifications in signal transduction pathways: Antidepressants have the ability to regulate intracellular signal transduction pathways that are important in the regulation of mood. Extended exposure to these medications can result in modifications in these pathways, which can impact the subsequent consequences of neurotransmitter signaling. Modifications in these pathways can contribute to the formation of tolerance by modifying the cellular reaction to serotonin and other neurotransmitters. (4) Enhanced neurotransmitter reuptake: Certain antidepressants function by impeding the reuptake of neurotransmitters such as serotonin, thus augmenting their concentrations in the brain. Nevertheless, during an extended period of use, the brain may adjust by enhancing the effectiveness of neurotransmitter reuptake systems. This can diminish the effectiveness of the antidepressant and contribute to the development of tolerance. (5) Neuroplasticity alterations: Extended exposure to antidepressants can induce modifications in neuroplasticity, which pertains to the brain's capacity to restructure its composition and operation in reaction to stimuli. These alterations can impact the brain's response to neurotransmitters and contribute to the formation of tolerance [19, 20].

Multiple clinical investigations have been undertaken to examine the emergence of drug tolerance towards different antidepressant medications. These research studies have investigated the mechanisms that cause tolerance, the factors that affect its formation, and methods to prevent or overcome it. Researchers have also investigated the therapeutic ramifications of drug tolerance, including its influence on treatment results and the necessity for dose modifications or prescription substitutions over time. Results from retrospective studies suggest that antidepressant treatment response patterns (late onset and durable vs. other patterns) can be utilized to forecast relapse during maintenance and continuation treatment, as well as potentially detect placebo effects.

After 6 months of continuous fluoxetine usage, a double-blind research of 517 depression patients found a relapse rate of 35.2%; after 1 year of follow-up, the percentage rose to 45.9%, suggesting that the efficacy of fluoxetine gradually decreased during the maintenance period [22]. Another study found that 60.4% of people who used antidepressants for a long time ended up experiencing a recurrence of their depression. This shows that although these medications help control depression, they may not be able to avoid recurrences for everyone [23]. Patients who have experienced a relapse while taking 20 mg of fluoxetine daily for an extended period of time respond well to a comparable medicine at a higher dose of 40 mg daily [24]. The studies also reported the development of tolerance to monoamine oxidase inhibitors and other antidepressant drugs which are found to be nonresponsive to the higher dose of the drugs indicating the existence of pharmacodynamics tolerance [19, 25]. The responsiveness to increased drug dosage for the treatment of patients of relapse during the maintenance period is also reported by a study. Though the study, on a 25-week trial, has also reported the rerelapse of the condition, in one out of 5 patients, on the continued use of the dose to which the patient responded on the first relapse [26]. Similar results of disease relapse were reported in a placebo-controlled study of the drug duloxetine on patients with major depressive disorder [27]. A meta-analysis performed on various studies on patients taking antidepressant drugs as a maintenance therapy reported an increased relapse risk which is found to increase progressively as 23% over 1-year usage, 34% over 2-year usage, and 45% over 3 years usage [28]. A study by the FDA adverse event reporting system reported a total of 60.79% of drug inefficacy cases and 39.21% occurrence of drug tolerance in patients of major depressive disorder on long-term usage of selective serotonin and serotonin–norepinephrine reuptake inhibitors [29].

### 2.2. Cancer

The presence of drug resistance and tolerance in cancer treatment is a complex issue that significantly hinders the effectiveness of pharmacological treatments. This is due to the quick remission of tumor cells, leading to a resurgence of the disease. The factors found to determine the evolution of drug resistance include the kinetics of tumor growth and burden, heterogeneity in tumor, the immune system, physical barriers, microenvironment of the body, undruggable cancer targets, and the outcomes of therapeutic pressures applied during the therapy. The extended use of anticancer medications leads to the development of drug tolerance, which includes intricate biological pathways. An important process involves the excessive production of drug efflux pumps, such as P-glycoprotein, which actively expel anticancer medications from cancer cells, thereby decreasing their concentration inside the cells and limiting their effectiveness. In addition, cancer cells can acquire resistance via modifying drug metabolism, such as by enhancing the function of drug-metabolizing enzymes and increasing the activity of drug detoxification pathways. Genetic mutations in drug targets can lead to resistance by decreasing the ability of drugs to attach to the target and limiting their effectiveness. In addition, cancer cells can stimulate survival pathways, such as the PI3K/AKT pathway, to enhance cell survival when exposed to anticancer medications. Alterations in the tumor microenvironment, such as heightened hypoxia or inflammation, can additionally contribute to the development of drug resistance. Epigenetic modifications, such as DNA methylation and histone modifications, can modify the patterns of gene expression and facilitate the development of drug tolerance. Gaining a comprehensive understanding of these molecular pathways is of utmost importance to devise strategies to combat drug resistance and enhance the efficacy of anticancer treatments [30]. Initially, polychemotherapy with drugs acting via different mechanisms emerged as a solution to the issue of drug resistance observed towards single-drug therapy and worked for some patients of lymphomas, testicular cancer, and breast cancer [31–34]. Various complex regimens with different dose intensities worked for years and ultimately failed [30]. The development of acquired resistance in cancer cells is a significant worry. This resistance might occur towards the initial therapy employed or against drugs from other classes that work through distinct pathways, resulting in cross-resistance [35]. A study also highlighted a new prospect on how nongenetically unstable tumor cells adapt to antiproliferative drugs when some selective forces act on them resulting in increased growth of rapidly multiplying drug-resistant cancerous cells [36]. Lysosomes are also reported to play a part in developing resistance towards various weakly basic lipophilic anticancer drugs. Since lysosomes and lysosomal proteins are hardly the target site of anticancer drugs, the weakly basic lipophilic drugs get sequestered in lysosomes and will not be accessible at the target site, thus reducing their cytotoxic effect as a lower concentration of the drug will reach the target site [37]. Another study attributed the development of multiple drug resistance in cancer patients to endolysosomal drug trafficking and suggested that selectively blocking the vacuolar H-ATPase might be an approach to overcome drug resistance to chemotherapy [38]. Shreds of evidence also support the existing irreversible proteomic and epigenetic mechanism in the emerging resistance to anticancer drugs. The microenvironment of tumors and the heterogeneity of cancer cells could also be responsible for the development of resistance in cancer cells [39]. In a study, exosomes were found to have a significant role in resistance development towards anticancer drugs. The exosomal proteins or noncoding RNAs present within the exosomes of tumor cells result in drug resistance by controlling the efflux of the drug, drug metabolism, prosurvival signaling, the transition of epithelial cell layer to mesenchyma, and remodeling the microenvironment of tumor cells [40]. Furthermore, significant anticancer effects have been observed *in vitro* and *in vivo* upon inhibition of Hsp90 activities, which impact numerous oncogenic substrates concurrently. The 17-allylamino-17-demethoxygeldanamycin (17-AAG) Hsp90 inhibitor has finished phase II clinical studies in multiple cancer types. Inhibitors of N-terminal Hsp90 that obstruct the ATP-binding domain of Hsp90 include geldanamycin and its variants and structurally distinct chemicals such as radicicol. Several inhibitors based on novobiocin and coumarin, as well as other C-terminal Hsp90 inhibitors, are undergoing preclinical research [41].

### 2.3. Inflammatory and Analgesic

NSAIDs are the most commonly used over-the-counter drugs and are used for prolonged duration in various chronic inflammatory conditions. Amongst the most prevailing adverse effects of this class is the development of acquired tolerance over prolonged use. Various studies are performed to describe the mechanism of occurrence of drug tolerance towards NSAIDs and it has been reported that the drug tolerance to NSAIDs follows the endogenous pathway of the opioid system probably involving the central pain modulatory system. The researchers have reported that the microinjection of NSAIDs in several areas of the midbrain, such as the amygdala, nucleus raphe, cerebral cortex, dorsal hippocampus, and gray matter, has the antinociceptive effect which can be inhibited or diminished with pretreatment or posttreatment with naloxone, an opioid antagonist; thus supporting the fact that drug tolerance to NSAIDs follows the endogenous pathway of the opioid system [42–46].

Opioid analgesics are also an important class of analgesics that is used over all other alternative therapies available for acute and chronic inflammatory diseases. The prolong use of these drugs also results in analgesic tolerance which is mostly conceived to be pharmacodynamic as the loss of analgesia is attributed to complex neuronal adaptations. The dose escalation is usually not considered as opioids have dose-dependent adverse effects such as opioid-induced hyperalgesia, so poor responsiveness to opioids at the maintenance dose is a major concern [47, 48]. The prolonged use of opioid analgesics decreases the release of GABA presynaptically and also decreases the presynaptic opioid inhibition within GABAergic nerve terminals. The GABAergic neurons are also inhibited postsynaptically on long-term use; thus the inhibiting G-protein–opioid receptor coupling inwardly rectifies K^+^ conductance and voltage-gated calcium channels within periaqueductal gray in the midbrain which is the major site of opioid action [49, 50]. Opioid therapy inhibits the production of cyclic AMP but on prolonged use, as the tolerance develops, cyclic AMP is upregulated. The stimulation of G-protein–opioid receptor couples and results in dissociate subunits (G*α* and G*βγ*) which then inhibits voltage-gated Ca^+^ channels and activates inward-rectifying K^+^ channels. Moreover, cyclic AMP levels are decreased due to inhibition of downstream adenylate cyclase enzymes. The decrease in neurotransmitter release in the midbrain periaqueductal gray is attributed to the activation of adenylyl cyclase–cyclic AMP-protein kinase A signaling pathway which is correspondent to antinociception. The *β*-arrestin, a predominantly expressed protein known for its part in desensitizing G-protein–coupled receptors, is recruited in response to opioid-mediated receptor phosphorylation by G-protein–coupled receptor kinase which desensitized the receptors due to receptor internalization [51, 52]. The diminished analgesic action, provoked intensity of pain, enhanced tolerance, and induced hyperalgesia are attributed to the factors such as stimulated adenylate cyclase activity, activated phosphokinase A, NMDA receptors, and downregulated glutamate receptors [53, 54].

### 2.4. Antibiotic Drug Resistance

Antibiotic drug resistance presents a disturbing and intensifying threat to clinicians. The prolonged use of antibiotics results in the development of resistant strains of the bacteria due to genetic mutations and horizontal gene transfer. The bacteria adopt some alternative methods of metabolism when acted upon by antibiotics for a longer duration as the antibiotics target the metabolic pathways of the bacteria. Though the bacteria are usually susceptible to the higher doses of the same antibiotic agent even after becoming resistant, however, they can withstand the effects of the drug but are unable to grow. The recurring infection is found to be caused mostly by the relapse of the original strain of the bacteria which has developed resistance and not the new mutated strain. Several studies on *in vivo* models have reported the development of tolerance by the slowly growing bacterial population [55]. The evolution of resistant bacteria in an *in vitro* laboratory setup is homogenous as the microtiter is usually used which results in the homogenous mixing of the drug in the culture media so all the bacteria are exposed to a uniform concentration of the drug, whereas in the real condition, the drug cannot be distributed uniformly owing to the difference in tissue permeability, target protein binding, and the drug inactivation. Hence, the evolution of resistant bacteria in a heterogeneous environment is practically achieved and nonuniformly available drug concentration results in the development of the more resistant strain of the bacteria [56]. Drug resistance results in mutations in the *marR* family repressor gene which thus fails to inhibit *AraC* family activators. The latter then communicates an enhanced expression of resistance nodulation division efflux pump *AcrB* and periplasmic adapter *AcrA* that enhances the efflux of the drug from the target cells [57]. Bacteria employ many mechanisms to evade the detrimental impacts of antibiotics, resulting in the emergence of resistant strains. The bacterial cells expel drugs to decrease their concentration below the therapeutic threshold. The antibiotic agents undergo chemical degradation by bacterial enzymes, resulting in their deactivation. The emergence of the aforementioned enzymes is a consequence of genetic mutations occurring within bacterial cells during the development of resistance. The modified drugs become inactivated as they are not recognized by the target binding site. The target sites of the antibiotics within the bacteria can be modified by rearranging the amide linkages to decrease their affinity for the antibiotic agents [58, 59].

Resistance to antifungal drugs has been a concern for clinicians in treating systemic infections. Many patients do not respond to pharmacological therapy even if prescribed with the drugs to which the infecting fungi are susceptible. This indicates the existence of tolerance to the antifungal drugs [60]. The fungal infections are mostly seen associated with the infectious diseases affecting/suppressing the immune system such as salmonellosis, tuberculosis (TB), and human immunodeficiency virus or in the patients who have undergone any organ transplantation procedure or cancer [3]. The azoles are the most commonly used antifungal drugs and resistance to azole is mainly acquired or it may be due to the selection pressure in the naturally resistant strains [61]. The slightly altered amino acid sequencing results in the development of the resistant strains, whereas major alterations in the sequencing may lead to the loss of protein function thus affecting metabolism which accumulates toxins [3]. The two major efflux systems found active in conferring resistance to azoles or other antifungal drugs consist of the proteins from the major facilitator superfamily (MFS) and ATP-binding cassette (ABC). The ABC transporter is reported to be majorly involved in the drug resistance mechanism. In the fluconazole-resistant *Candida* strains, MDR1 is found to be responsible for encoding the resistance for methotrexate and benomyl also. Drug resistance in *S. cerevisiae* is found to be attributed to ABC transporters grouped in five families, namely, CFTR, MDR, PDR, and YEF. A total of five genes are found responsible for developing resistance against azoles in *Candida* species known as *Candida* drug resistance genes (CDR genes) [3]. Overexpression of genes, such as CDR1 and ABC transporter, is seen with reduced accumulation of azole drugs such as fluconazole in a study on *Candida albicans*. The prolonged use of azole drugs is also believed to cause induction of ERG16 and CDR1 genes, hence resulting in cross-resistance amongst the azole class of antifungal drugs [3]. Factors such as Hsp90, target of rapamycin (TOR), Rim 101 mutants (a transcriptional factor), calcineurin, and lysine deacetylase are found to contribute to the development of drug tolerance to antifungal drugs and disruption/elimination of all these factors result in reversal to tolerance thus reversing drug resistance [62, 63]. The mutation in the ERG genes is also found associated with the existence of drug resistance in various *Candida* species [64].

Similar to bacteria and fungi, certain viruses have the ability to rapidly reproduce even when exposed to specific antiviral medications over a lengthy period of time. This leads to the emergence of resistant variants within the population [65]. The antiviral drugs are used to act and interfere with the growth of viruses by attacking the mechanisms involved in their replication process. If the treatment is noneffective or less effective, some viral genomes will get a chance to grow even under selective pressure that will result in the growth of mutant strains of viruses [66]. Hepatitis C virus (HCV) is known for its excessive mutation ability and genomic variation [67]. The higher mutation rate is attributed to recurrent replication and lack of proofreading by viral RNA polymerase. Antiviral drugs used to treat HCV act by inhibiting the activity of enzymes, protease or polymerase. Moreover, protease resistance confronts the least barriers and so, is easy to be achieved within a few mutations [68]. Combination therapy can be a better alternative for the treatment as it offers a strong barrier to resistance and also cross-resistance is not commonly reported with combination therapies [69]. Influenza A virus, an RNA virus, codes for around eleven proteins. The two proteins, hemagglutinin and neuraminidase are present on the surface of the virus and facilitate viral attachment and detachment, respectively, to the target cell membrane. These two surface proteins are reported to have higher mutation rates as they are exposed to a diverse environment and selection pressure [70, 71]. Herpes simplex virus is found to develop resistance to acyclovir via mutations in tyrosine kinase or DNA polymerase. The mutations in tyrosine kinase are more common and are the result of altered guanine–cytosine homopolymeric runs. Cross-resistance for penciclovir and ganciclovir is seen in tyrosine kinase–deficient-acyclovir-resistant mutants [72]. HIV encodes from the RNA genome when in virion but once it enters the host cell, it replicates using reverse transcription resulting in double-stranded DNA. As reverse transcriptase is reported to be highly prone to errors, this makes it an easy target for mutation. Hence, HIV is always treated with combination therapy including drugs from a different class of drugs that lack cross-resistance [73, 74].

### 2.5. TB

TB is one of the few diseases, the treatment therapy of which usually fails if not adhered to completely. The major reason attributed to the failure of the therapy is the development of resistance towards one or more of the first- or second-line drugs [75]. An important process is the occurrence of genetic mutations in the genes responsible for pharmacological targets or enzymes involved in drug activation or inactivation. For instance, alterations in the genes that encode the mycobacterial DNA gyrase or RNA polymerase can result in resistance to fluoroquinolones or rifampicin, respectively. For example, *Mtb* encodes the β-lactamase enzyme so presents resistance to *β*-lactam antibiotics. The prolonged use of antitubercular drugs leads to nonadherence to the therapy, thus rapidly mutating *Mtb* leads to drug resistance [76–79]. Mycobacteria take advantage of their dormant state to survive in the host and develop into the resistant population. They like other bacteria, make use of the entire efflux system for survival. *M. TB* like other bacteria gives rise to the resistant strains by mutating the genes responsible for the efflux system and cell wall permeability. Gene mutations also play a significant role in the development of drug resistance towards anticancer drugs. Mutations in the genes *rrs* and *rpsL* give rise to streptomycin resistance altering the drug's target ribosomal binding site. Pyrazinamide resistance results due to altered *pncA* gene and isoniazid are aimed by attacking the drug targets contributing to cell wall synthesis [3]. The tolerance to the antitubercular drugs develops the selective pressure of pharmacological therapy either by metabolic shift or efflux pumps or altered cell wall permeability. The metabolic shift is associated with the decreased TCA cycle resulting in lipid synthesis and thus The metabolic shift is associated with the decreased TCA cycle which results in more lipid synthesis which increases the thickness of the cell wall, hence decreasing its permeability and also its susceptibility towards antitubercular drugs Similarly, prolonged use of rifampicin also results in drug tolerance by overly expressed drug target *rpoB* [80].

### 2.6. Failure of Drug Therapy

Pharmacological interventions for various ailments which require prolonged use of drug/drugs usually fail after some time. The treatment of diabetes mellitus fails when the blood glucose level is not maintained by oral hypoglycemics which then require either higher doses of the same drug or a combination of drugs [81, 82]. After a few years of starting therapy with sulfonylureas, patients eventually transition to needing daily insulin to regulate blood glucose levels. This is because sulfonylureas stimulate the *β*-cells of the pancreas excessively, leading to the death of these cells (*β*-cell apoptosis) [83]. Hypertension is also a disorder that requires lifestyle changes and prolonged pharmacological interventions. Resistant hypertension develops on prolonged usage of the drug and is defined as the treatment failure with more than 3 subclasses of antihypertensive drugs [84]. Rheumatoid arthritis treatment also fails on prolonged usage of the disease-modifying drugs and one amongst the several reasons for this is reported to be the loss of the efficacy of the drug which is initially effective and drug resistance [85]. Some medications have less of an impact and the likelihood of drug resistance increases in those with vitamin D insufficiency. To explain this occurrence, various explanations have been suggested. Due to its role in immune system modulation, vitamin D deficiency can reduce the body's ability to ward off infections and malignancies. Vitamin D also affects the effectiveness of some medications by regulating the expression of genes that are involved in drug metabolism and transport. In addition, chronic inflammation, which vitamin D insufficiency is associated with, might foster drug resistance by providing an ideal setting in which drug-resistant cells can flourish. In general, keeping vitamin D levels adequate may lessen the likelihood of drug resistance and increase the efficacy of medication therapy. Vitamin D finds its role in the treatment of various metabolic disorders, cardiovascular diseases, autoimmune diseases, tumors, granuloma-forming disorders, etc. as adjunctive therapy [86].

## 3. Role of Vitamin D in Overcoming Resistance

**Figure 1:**
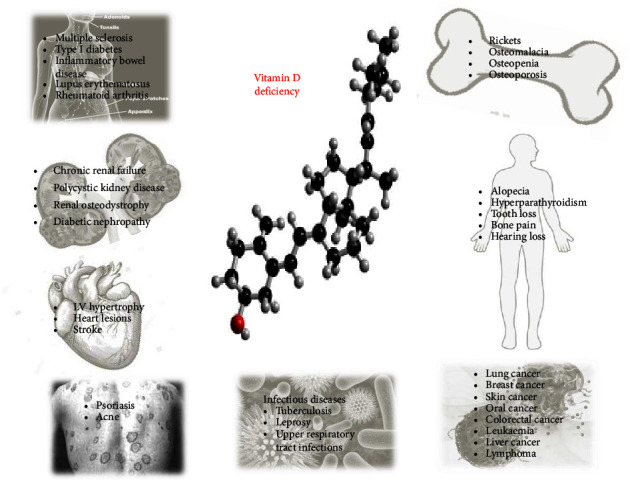
Vitamin D deficiency association in the pathogenesis of various diseases.

Vitamin D, the sunshine vitamin is a necessary fat-soluble vitamin required by the body for the absorption of calcium and helps to prevent various bone-associated abnormalities. Though vitamin D can be synthesized by the body using ultraviolet radiation of sunlight, still vitamin D deficiency is a major health concern of today owing to the changing lifestyle. Vitamin D deficiency is, however, associated with many diseases (Figure 1), still it cannot be conceived that replenishing the stores of vitamin D in the human body can confer better clinical outcomes and survival [87].

Vitamin D occurs in 2 different forms, namely, ergocalciferol and cholecalciferol. While ergocalciferol also known as vitamin D_2_ is found in plants and some fishes, cholecalciferol also known as vitamin D_3_ is synthesized in the body, i.e., epidermis of the skin on the exposure to UVB rays where 7-dehydrocholesterol is photoconverted to previtamin D_3_ which is then isomerized to form vitamin D_3_. Both the forms of vitamin D_3_ are metabolized by the enzyme 25-hydroxylase in the liver to synthesize the inactive precursor of vitamin D, i.e., 25-hydroxy vitamin D [14]. Cholecalciferol acts as a precursor for the calcitriol hormone. The substrate 7-dehydrocholesterol is converted to vitamin D_3_ in the skin in the presence of ultraviolet rays which is then hydroxylated to calcitriol (1,25(OH)_2_D_3_) in cytochrome P450. The hydroxylation reaction yielding calcitriol is a 2 step process: the first hydroxylation occurs in the liver and yields 25(OH)D_3_ on catalyzation by vitamin D 25-hydroxylase (mostly CYP2R1) and the second step takes place in the kidney, where hydroxylation of 25(OH)D_3_ occurs at C1*α* position by CYP27B1 (1*α*-hydroxylase), thus producing calcitriol/vitamin D_3_/1,25(OH)_2_D [88]. In the kidneys, the key signal for triggering CYP27B1 transcription is the parathyroid hormone (PTH). The parathyroid glands respond to the lower serum levels of calcium by releasing PTH, thus enhancing vitamin D_3_ synthesis by increasing the expression of CYP27B1. The CYP27B1 is also overexpressed in response to decreased phosphate levels in the blood. The released vitamin D_3_ enhances the absorption of calcium and phosphate from the intestine and also increases receptor activator of nuclear factor-*κ*B ligand (RANKL) along with PTH resulting in the stimulation of giant osteoclasts to trigger bone resorption [89].

Calcitriol acts by binding and phosphorylating the nuclear vitamin D receptor (VDR). VDR is found in almost every cell of the body and calcitriol can regulate either directly or indirectly around 3%–5% of the human genome. Vitamin D can also affect the defense system of the body, hence limiting the pathogenesis of several diseases [90, 91]. Calcitriol is an autoregulated enzyme and induces 24-hydroxylase or CYP24A1 to degrade 1,25(OH)_2_D and 25(OH)D [92]. Calcitriol is also found to regulate renal CYP27B1 and CYP24A1. CYP27B1, though majorly present in the kidney is also found to expressed in several extrarenal sites such as cancer cells, which suggests the role of dietary vitamin D in cancer therapy [87, 93]. An *in vitro* study on leukemic Jurkat/ADR and K562/ADR cell lines has reported the reversal of drug resistance after treatment with vitamin D in a dose-dependent manner. Vitamin D successfully diminished the expression of multiple drug–resistant genes (MDR1) and multiple drug resistance–related genes (MRP1). The cell wall content of P-glycoprotein and intracellular levels of glutathione are also found reduced. The resistance here is believed to be due to decreased MDR 1 and MRP1 gene expression which in turn blocks the GSH efflux pump that is responsible for throwing the drug out of the cancer cells [94].

### 3.1. Depression

Various studies have reported the association of serum vitamin D deficiency with moderate and severe depressive disorder. Vitamin D supplementation is found to play a crucial role in ameliorating the symptoms of mood and depressive disorder. The role of vitamin D in the brain is still not clear but it is believed to enhance tyrosine hydroxylase gene expression and also the bioavailable levels of dopamine, norepinephrine, and epinephrine were found to increase on vitamin D supplementation. In a complex process including gene regulation, neuroprotection, anti-inflammatory activities, and modulation of neurotransmitter systems, vitamin D and its receptors are essential in reversing antidepressant medication resistance. In order to regulate mood and the effectiveness of antidepressant medications, the active form of vitamin D, calcitriol, attaches to VDRs in the brain. This influences the production and release of important neurotransmitters such as serotonin, dopamine, and norepinephrine. By regulating the transcription of many genes, activation of VDR promotes neurogenesis and increases neural plasticity. The anti-inflammatory actions of vitamin D, lower levels of proinflammatory cytokines, and raised levels of anti-inflammatory cytokines make the brain more receptive to the antidepressant therapeutic effects. Vitamin D also improves the brain's reaction to antidepressants by increasing the expression of BDNF, a protein that helps neurons grow, survive, and differentiate. Vitamin D enhances the bioavailability and effectiveness of antidepressants by downregulating enzymes that metabolize them. In sum, the brain becomes more receptive to antidepressant treatment as a result of these interrelated processes, which aid in the fight against medication resistance.

**Figure 2:**
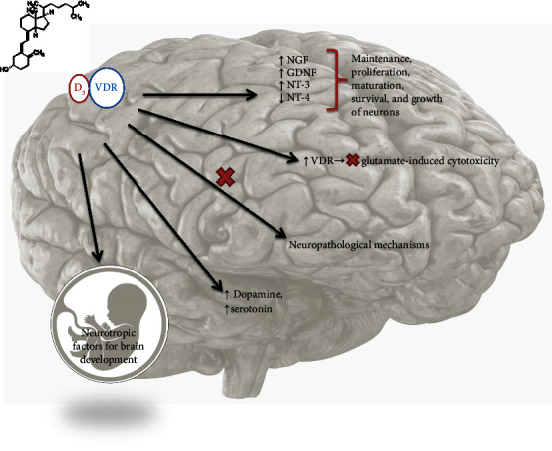
Vitamin D affecting the pathophysiology of major depression and brain development.

Calcitriol is also found to have the potential of aggravating the levels of some neurotrophic factors having a significant role in depression-like nerve growth factors, glial-derived neurotrophic factors, and Neurotrophin-3 (Figure 2). In the same study, fluoxetine when combined with vitamin D supplements is found to better control the symptoms of depression in comparison to fluoxetine administered alone [95]. The systematic review and meta-analysis have proven the advantages of vitamin D supplementation in individuals diagnosed with vitamin D deficiency [96, 97]. Observational studies conducted on humans, which subsequently advanced to clinical trials, have indicated that vitamin D insufficiency is likely linked to illness progression and also plays a role in the disease's pathophysiology [98, 99]. In a study on elderly patients with depression, tricyclic antidepressants were found to reduce the levels of 1,25-(OH)_2_ vitamin D3, and thus 1,25-(OH)_2_ form of vitamin D3 is believed to have a role in the etiology of the disease and late-life depression [100]. A study including mild to moderate depression patients administered with 50,000 IU/2-weeks vitamin D supplementation reported an increase in the concentration of 25(OH)D and reported the amelioration of severe symptoms of depression [101].

In another clinical trial, researchers studied 355 older individuals with depression and 124 nondepressed individuals of the same age. The results showed that tricyclic antidepressants decreased the levels of 1,25-(OH)2 vitamin D3 but did not have an effect on its precursor, 25-OH vitamin D3, which is commonly measured. The study thus determined the causal function of vitamin D in depression that occurs in later stages of life [100]. The findings of another 8-week randomized clinical trial involving 56 persons who had mild to moderate depression, with an average age of 43.0 ± 1.15 years, revealed that supplementing with 50,000 IU/2 weeks of vitamin D increased the concentration of serum 25(OH)D in the participants and considerably decreased the severity of their depression, thus clearly indicating the significance of vitamin D in the development of the disease and depression in older adults [101].

### 3.2. Cancer

Vitamin D is reported to play a part in antitumor pathways by inciting differentiation of cells, impeding cellular proliferation, modulating the impression of oncogenes and immunity, and hence controlling tumor cell cycle and actuating cell apoptosis. Overexpressed in drug-resistant cancer cells, drug efflux pumps like P-glycoprotein actively expel anticancer medications from the cells, diminishing their effectiveness. The downregulation of these pumps is a critical factor in this process. Vitamin D enhances the efficacy of anticancer medications by raising their intracellular concentration by blocking these pumps. Vitamin D also makes the tumor microenvironment more receptive to treatment by lowering inflammation and changing the immune response, which makes the environment less favorable for cancer growth. In addition to increasing the cytotoxic effects of anticancer medications, it causes apoptosis in cancer cells by downregulating antiapoptotic genes and upregulating proapoptotic genes. Vitamin D also promotes differentiation and suppresses cancer cell growth, which lessens tumor aggressiveness [102, 103]. In a study, vitamin D is also found to enhance the susceptibility of tumor cells to chemotherapy and have a synergistic effect when used in combination with anticancer drugs in the treatment of lung cancer [94]. The lower concentration of anticancer drugs is more effective and less harmful when used in combination with vitamin D [104].

**Figure 3:**
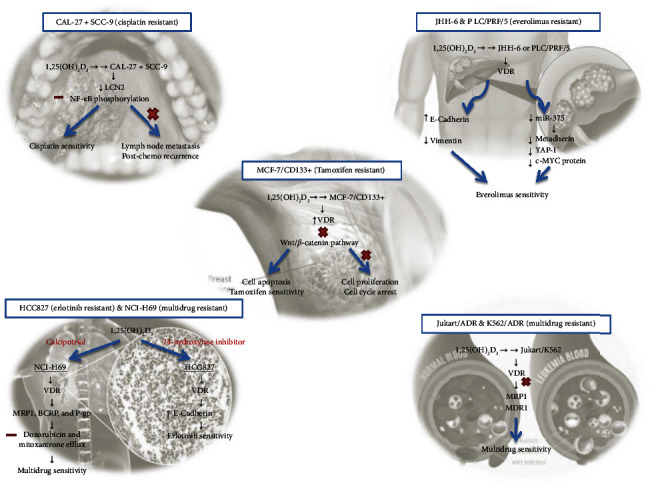
Role of Vitamin D in reversing anticancer drug resistance mechanisms.

A study reported the anticancer effect of vitamin D and suggested that calcitriol exerts a protective effect on cancer cells by dimerizing with retinoid X receptor in a bound form with VDR, and then translocating to the nucleus. The promoters of target genes have multiple regulatory genes wherein VDR–RXR binds to vitamin D response elements (VDREs). The coactivators or copressors are recruited with the VDR–RXR–VDRE complex which then results in the transcriptional regulation of gene expression [88]. The preventive effect is attributed to the role of the vitamin in transcriptional modulation which results in inhibited cellular proliferation and angiogenesis, enabling cell differentiation and apoptosis by interfering with the signaling pathways of numerous growth factor–activated receptors [105]. In one more study, the investigators interpreted the role of several target genes associated with vitamin D that play a crucial role in hampering tumorigenesis in hepatocellular carcinoma [106–108]. Vitamin D is also found to regulate the microenvironment of tumor cells and thus facilitate tumor repression [16]. In a similar line of research, a clinical trial was conducted on 25,871 participants to assess the impact of vitamin D on the progression of cancer. The trial was randomized, double-blind, and placebo-controlled. The results showed that the use of vitamin D as an additional therapy significantly reduced the occurrence of advanced cancers (metastatic or fatal) compared to the use of a placebo. When categorized based on BMI, there was a notable decrease in the occurrence of metastatic or deadly cancer among individuals with a normal BMI (BMI < 25) who were assigned to the vitamin D group. However, this reduction was not observed among individuals with overweight or obesity (BMI ≥ 30) [109]. Vitamin D provides an additive antiproliferative effect when used as an adjuvant. It, owing to its anti-inflammatory effect, is found to reduce the severity of cancer. In numerous animal studies in colorectal tumors, the combination therapy of vitamin D and 5-fluorouracil (5-FU), is reported to show an augmented protective effect in comparison to 5-FU used alone [110–115]. Also, dietary vitamin D and calcitriol are reported to have a similar intensity of anticancer effect in animal models of breast and prostate cancer [116]. In some *in vitro* cancer models, vitamin D is documented to reverse the mechanism of drug resistance to chemotherapy [117, 118], tyrosine kinase inhibitors [119], and a few targeted therapies [94, 120, 121] (Figure 3).

### 3.3. TB

Reversing medication resistance to TB is a major function of vitamin D and its receptors, which exert antibacterial and immunomodulatory actions. Calcitriol, the active form of vitamin D, strengthens the immune system's capacity to fight *Mycobacterium TB* by binding to the VDR on cells including macrophages and T-cells. To directly kill *Mycobacterium TB*, VDR activates genes involved in the immune response, increasing the production of antimicrobial peptides such as defensins and cathelicidin. Furthermore, vitamin D improves the efficiency of anti-TB medications by increasing the autophagy pathway in macrophages, a cellular process that breaks down and recycles intracellular infections. In addition to lowering inflammation, vitamin D decreases the production of proinflammatory cytokines involved in tissue damage and the ineffectiveness of pharmacological treatments. Vitamin D improves the efficacy of anti-TB medications by lowering inflammation and regulating the immunological response. In addition, vitamin D's ability to boost the immune system as a whole means that more bacteria may be eradicated during TB therapy, which in turn helps to avoid the development of drug-resistant strains. A study conducted by AIIMS and RBIPMT hospital in New Delhi examined 897 subjects from northern India to investigate the relationship between VDR polymorphisms and serum 25(OH)D levels with susceptibility to, and response to treatment of, multidrug-resistant TB (MDR-TB) in comparison to drug-susceptible pulmonary TB (DS-PTB) and healthy controls. The findings indicated that decreased levels of vitamin D and genetic variation in the VDR are associated with drug resistance in pulmonary TB. This is further supported by an additional finding from the study which found that lower serum levels of vitamin D prolong the duration of sputum smear negativity in MDR-TB [122]. VDR gene polymorphs are found to influence the immunomodulatory effect of vitamin D. Apal, Bsml, Fokl, and Taql are the four single nucleotide polymorphs of the VDR gene that are found to affect host susceptibility to TB. In a meta-analysis, the VDR gene variants which are homozygous for Fokl are reported to be more susceptible to TB; the Bsml variant may have a protective effect [123], whereas, in another study, Fokl polymorphism is not found to be responsible for enhancing the host susceptibility to *M. TB* [124]. Another case-control study comparing the vitamin D levels of individuals with pulmonary MDR-TB (including XDR and pre-XDR) to those without the disease, who are the residents of Mumbai and aged between 18 and 60 years, has indicated a potential avenue for further research to explore the impact of vitamin D supplementation in combating drug resistance, including multiple drug resistance [125]. Moreover, a meta-analysis of 23 relevant studies revealed that individuals with the Fokl ff genotype exhibited a strong positive association with TB, while those with the Bsml bb genotype showed a significant inverse association. In addition, marginal significant associations were found for the Taql and Apal polymorphisms among Asians. These findings suggest that the risk of developing TB is elevated in individuals with specific variations in the VDR gene, highlighting the potential role of vitamin D in the development and progression of TB [126].

### 3.4. Microbial Infections

Vitamin D is proven to have antimicrobial and immune-modulatory potential in an optimal concentration which may vary with different microbial infections. VDRs on immune cells such as macrophages, monocytes, and T-cells bind to calcitriol. Due to this interaction, genes that are involved in the immune response are transcribed, and peptides with broad-spectrum antimicrobial activity against bacteria, viruses, and fungi are produced, for example, defensins and cathelicidin. These peptides increase the efficacy of antimicrobial medications by destroying microbes and penetrating their membranes. Vitamin D also improves the efficiency of antimicrobial treatment by increasing immune cell autophagy, which leads to a decrease in microbial burden through the breakdown and recycling of intracellular infections. In addition, vitamin D regulates the inflammatory response by reducing levels of proinflammatory cytokines, which could lead to tissue damage and make it harder for antimicrobial medications to work. Vitamin D may also affect the production of metabolic enzymes and drug efflux pumps, which may lead to an increase in intracellular concentration of antimicrobial drugs and a decrease in efflux, two factors that contribute to the development of resistance to microbes [127].

Vitamin D has widely ranging systemic actions such as enhancing the generation of AMPs which is of benefit to the host in combating microbial growth except for leishmaniasis. Vitamin D is reported to have a protective role in various acute and chronic respiratory tract infections, influenza, herpes, HIV, hepatitis, etc., as its levels are found to decrease in all the mentioned viral infections. *In vitro* studies also supported that vitamin D is found to have the inhibitory potential for the bacterial strains, namely, *S. aureus, S. pyogenes, K. pnuemoniae, E. coli,* and several other bacteria [123]. Vitamin D is reported to be a potential molecule in combating the various Gram-negative and positive bacterial growth and MDR strains. Vitamin D alone or as an adjunctive agent with antibiotic drugs is found useful in some studies; however, a few conflicting outcomes are also obtained in some *in vivo* studies [128].

Vitamin D plays a role in combating various infections caused by fungi. It is found to increase the circulating natural killer cells which enhance and trigger the host immune system to fight against fungal infections [129].

Though there is a lack of data supporting the effect of vitamin D in parasitic infections, few *in vitro* studies found the toxic degenerative effects of vitamin D *Hymenolepis microstoma* [123]. Another clinical study on children for helminth infection reported a decrease in the rate of recurrence of infection with *Schistosoma mansoni* [130]. Consistent with previous studies on helminth infections, a randomized, placebo-controlled, double-blind, two-by-two factorial experiment including 977 students from 19 primary schools in Kenya found that the incidence of *Schistosoma mansoni* recurrence was lower [129].

## 4. Conclusion

Various studies have been conducted to date which report and support the role of vitamin D in the etiology of several chronic disorders. Where the deficiency of vitamin D is found to increase the risk of worsening of the disease, supplementation with adequate doses of the vitamin is found to ameliorate the symptoms associated with the chronic disorder. Vitamin D is found to be effective in preventing the pathogenesis of depression and various studies have supported its presence and role in the pathophysiology of major depression. Also, its role as an adjuvant to the drugs such as fluoxetine is proved by the studies, so we believe and suggest exploring its role in reversing drug tolerance. Moreover, various types of cancerous cell lines with resistance to one or the other anticancer drugs have proved to regain sensitivity for the drugs when treated with vitamin D. Some studies also suspect the role of vitamin D in TB and several microbial infections and opens the way to further research studies to investigate the reversal of drug resistance. One of the most important challenges faced by clinicians in the pharmacological treatment of chronic ailments is the long-term use of the drugs which possibly leads to drug resistance and tolerance and hence failure of the drug therapy. Also, vitamin D deficiency is associated with almost every chronic disorder; so it can be concluded and hypothesized from the abovementioned studies that vitamin D may also find its role in preventing the occurrence or reversing the mechanism of drug resistance and tolerance. The studies also reported the superiority of pharmacological treatment when combined with vitamin D supplements in comparison to the drugs administered alone. Further high-quality research is required to find the role of vitamin D supplementation in preventing drug resistance and tolerance.

There are a number of options to investigate in order to better understand how vitamin D works therapeutically to fight drug resistance and to choose the best way to use it. One possible experimental strategy is to use cell lines *in vitro* to study the molecular mechanisms, such as how vitamin D affects drug efflux pumps or drug metabolic pathways. Th*e in vivo* benefits of vitamin D could be further investigated through animal experiments that utilize models of drug-resistant illnesses. Vitamin D supplementation in patients undergoing conventional pharmacological treatments and studies examining the advantages of vitamin D in conjunction with current treatments are two examples of crucial clinical trials that must be conducted. Pharmacogenomic research has the potential to uncover genetic markers that indicate how a person will react to vitamin D, which could lead to more targeted approaches to treatment. Utilizing patient samples to investigate vitamin D metabolism and receptor expression, translational research might center on gaining molecular knowledge of vitamin D's impact on drug-resistant illnesses. If these methods work together, we may learn more about vitamin D's function in fighting drug resistance and find better ways to use it therapeutically in the clinic.

## Data Availability

The data used to support the findings of this study are included within the article.
